# Gender differences in first episode psychotic mania

**DOI:** 10.1186/1471-244X-13-82

**Published:** 2013-03-13

**Authors:** Sue M Cotton, Martin Lambert, Michael Berk, Benno G Schimmelmann, Felicity J Butselaar, Patrick D McGorry, Philippe Conus

**Affiliations:** 1 Orygen Youth Health Research Centre, University of Melbourne, Locked Bag 10 (35 Poplar Road), Parkville, Victoria, 3052, Australia; 2 Centre for Youth Mental Health, The University of Melbourne, Parkville, Victoria, Australia; 3 Psychosis Early Detection and Intervention Centre (PEDIC), Department for Psychiatry and Psychotherapy, Centre for Psychosocial Medicine, University Medical Center Hamburg-Eppendorf, Hamburg, Germany; 4 School of Medicine, Deakin University, Geelong, Australia; 5 Florey Institute for Neuroscience and Mental Health, Parkville, Australia; 6 University Hospital of Child and Adolescent Psychiatry, University of Bern, Bern, Switzerland; 7 Treatment and Early Intervention in Psychosis Program (TIPP), Département de Psychiatrie CHUV, Université de Lausanne, Clinique de Cery, Prilly, Switzerland

**Keywords:** Gender, Mania, Psychosis, Bipolar disorder

## Abstract

**Background:**

The aim of this paper was to delineate the impact of gender on premorbid history, onset, and 18 month outcomes of first episode psychotic mania (FEPM) patients.

**Methods:**

Medical file audit assessment of 118 (male = 71; female = 47) patients with FEPM aged 15 to 29 years was undertaken on clinical and functional measures.

**Results:**

Males with FEPM had increased likelihood of substance use (*OR* = 13.41, *p <*.001) and forensic issues (*OR* = 4.71, *p* = .008), whereas females were more likely to have history of sexual abuse trauma (*OR* = 7.12, *p* = .001). At service entry, males were more likely to be using substances, especially cannabis (*OR* = 2.15, *p* = .047), had more severe illness (*OR* = 1.72, *p* = .037), and poorer functioning (*OR* = 0.96, *p* = .045). During treatment males were more likely to decrease substance use (*OR* = 5.34, *p* = .008) and were more likely to be living with family (*OR* = 4.30, *p* = .009). There were no gender differences in age of onset, psychopathology or functioning at discharge.

**Conclusions:**

Clinically meaningful gender differences in FEPM were driven by risk factors possibly associated with poor outcome. For males, substance use might be associated with poorer clinical presentation and functioning. In females with FEPM, the impact of sexual trauma on illness course warrants further consideration.

## Background

Gender differences in incidence, pre-illness characteristics, onset and course have been widely documented in psychotic disorders such as schizophrenia [1-4]. Less is known regarding gender differences in bipolar I disorder. There are epidemiological studies indicating substantive gender differences in the incidence, presentation and course of bipolar I disorder; however, the extent of these differences is yet to be fully delineated. An equal gender ratio has been reported for bipolar I disorders [5]; however, other studies indicate that males may be more likely to be at risk of bipolar I disorder [6], or conversely that there is a higher incidence of bipolar I disorder in females [7].

There have been inconsistencies regarding age of illness onset for bipolar I disorder with some studies reporting absence of gender differences in age of onset [8], while others have documented an earlier onset in females [9] or an earlier onset in males [7,10]. The age range of patients included in cohorts is an important issue. Gender differences in age of onset of bipolar disorder may vary depending on the decade under investigation. Between 16–25 years of age, bipolar disorder may be more common in males, whereas across the other decades, females with bipolar disorder may predominate [7,11]. Developmental variations across the lifespan including cortical maturation, psychosocial development, and changes in the female reproductive cycle [10], need to be considered in the context of such findings.

Reported gender differences in course of illness have also been varied. In terms of symptomatology, females may have more severe depressive symptoms [9,10,12], more mixed symptoms [13], psychotic symptoms including paranoid delusions and delusions of reference [12], and be more likely to experience rapid cycling [9]. Other studies have found no such differences [8,14]. Polarity of the incipient mood episode may also contribute to variation in gender differences. Males with bipolar I disorder may be more likely to have mania as their first episode whereas females are more likely to have a depressive episode [8].

Less is known about gender differences in social and occupational functioning, with more emphasis being placed on symptomatology. However, Miquel et al. [15] reported that a higher proportion of females than males with acute mania, either lived independently or resided with a partner. No gender differences were observed in participation in work or social activities.

Unrepresentative and small samples may contribute to the variability in findings on gender differences in bipolar disorder. Often chronic patients or inpatients have been recruited; such cohorts have experienced a broad range of primary stressors prior to illness as well as secondary stressors associated with the illness (e.g., acute illness phases, relapses and medication).

To date, research on the gender differences in patients with first episode mania (FEM) is sparse. There appears to be only one study that has involved the investigation of gender differences in a FEM cohort. Kennedy et al., [7] focused on incidence across a wide age range (16 to 76 years+) and identified all adult cases with bipolar I disorder and FEM who had been in contact with services in a defined catchment area in south east London. They also considered a range of psychosocial factors that may relate to gender differences in age of onset. Males with FEM had an earlier onset disorder than females, and were also more likely to have a history of antisocial behaviour in childhood [7].

None of the studies conducted to date have examined gender differences in the subgroup of patients who have first episode psychotic mania (FEPM). Indeed, approximately 50% of patients will have psychotic symptoms at some stage of their illness [16]. Further, as around 70% of clients with bipolar disorder have incipient symptoms before the age of 25 years [17], it is important to consider whether there are gender differences in young adults with FEPM in terms of premorbid histories, entry characteristics, treatment, and discharge characteristics.

Therefore, in the current study, we report on gender differences in illness course of an epidemiologically representative treated cohort of patients with FEPM, aged between 15–29 years of age. It was hypothesized that there would be gender differences in clinical and functional characteristics of patients presenting with FEPM.

## Method

### Sample and setting

One hundred and eighteen patients with a discharge diagnosis of FEPM were selected from a larger cohort of patients with first episode psychosis (FEP) from the First Episode Psychosis Outcome Study (FEPOS). FEPOS comprised a file audit of all 786 FEP patients treated at the Early Psychosis Prevention and Intervention Centre (EPPIC) between January 1998 and December 2000 [1,18-27]. EPPIC provides a comprehensive specialist program for young people aged 15–29 years experiencing their first episode of psychosis. Its catchment area covers the north-western regions of Melbourne, Australia [28]. At the time of the study, EPPIC treated most patients with psychosis, with very little leakage into the private system [20]. On the basis of these characteristics, FEPOS has been considered to comprise a treated epidemiological cohort [20].

Institutional ethics approval was obtained from Mental Health Research and Ethics Committee, Melbourne Health, Victoria, Australia. As this was a medical file audit study, informed consent was not required to be obtained from patients.

### Materials and procedure

The Early Psychosis File Questionnaire [20] was used to systematically assess consecutive medical files.

Clinical diagnoses at EPPIC are derived through consensus during an intensive diagnostic and treatment process. An initial diagnosis is determined during the first 6 weeks of admission by the team of well-trained clinicians, and is reviewed over the course of treatment. FEPOS diagnoses were given according to DSM-IV and on the basis of all information detailed in the medical file [20]. Given the issues with diagnostic instability in early illness course, we used final discharge diagnosis to identify the cohort of FEPM patients used in the current study [23].

Pre-morbid characteristics recorded included: past history of DSM-IV psychiatric disorder; past history of DSM-IV substance use disorder; suicide attempts (classified on ICD-10 criteria); family history of psychiatric disorder; premorbid adjustment as assessed by the Global Assessment of Functioning (GAF); age at onset of psychotic symptoms; and duration of untreated psychosis (age of entry into EPPIC subtracted by age when first sustained positive psychotic symptoms longer than a week duration).

At service entry, clinical diagnoses (including substance use) were ascertained according to DSM-IV criteria. Illness severity was assessed according to scores on the Clinical Global Impressions–Severity of Illness Scale [29] and the Clinical Global Impressions-Bipolar Illness (CGI-BP). Insight was coded on a three point scale (0 ‘absence of insight; 1 ‘partial insight–perception of being ill but persistence of illogical explanations’; and 2 ‘full insight’).

Global functioning was assessed using the GAF. Vocational functioning was determined using the Modified Vocational Status Index (MVSI) and the Modified Location Code Index (MLCI) was used to determine independent living status [30].

Treatment information included: suicide attempts; inpatient admissions; medication non-compliance (≥1 week without taking medication); and service engagement.

At service discharge, the final file DSM-IV clinical diagnoses were recorded. Symptom severity was determined using CGI-S, CGI-BP, and the insight scale. The GAF, MVSI, and MLCI, were used to assess discharge functional status. Patterns of substance use over treatment was also documented and characterised by three categories: (i) no SUD; (ii) decreased or remitted (decrease in quality and frequency of ≥50% or remission of baseline at 18 months or time of discharge); and (iii) persistent SUD (either increased SUD or unchanged SUD) [20].

### Reliability and validity

Estimates obtained for inter-rater reliability for the CGI, CGI-BP, GAF, and insight for 40 files were good (range: *κ* = 0.87 for CGI-S to *κ* = 0.89 for insight score) [20]. The SCID-I/P was used to determine the validity of diagnoses for a subset of 115 patients (see Conus et al., 2007). There was good concordance for both psychotic (*κ* = 0.80) and substance use (*κ* = 0.74) diagnoses.

### Data analysis

A series of logistic regression analyses were conducted with gender as the dependent variable (male as the reference category), and the individual premorbid and service entry variables as predictors. From these analyses, odds ratios (*OR*) and the 95% confidence intervals (CI) of the *OR*s were derived. The Wald statistic (z) was used to determine significance of predictors. For the treatment and discharge variables, adjusted *OR*s and 95% CI of the adjusted *OR*s were reported, controlling for entry characteristics and time in service.

Multivariate logistic regression with forward stepwise variable selection (based on the likelihood ratio) was used to determine which factors best differentiated males and females with FEPM. Variables included in the equation were those that were different by gender in the univariate analyses at the *p* < .100 level.

Given the exploratory nature of the study, alpha (α) was set at .05 for all analyses. No adjustments were made for multiple comparisons because they can result in a higher type II rate, reduced power, and increased likelihood of missing important findings (Rothman, 1990).

## Results

The majority of patients were male (60.2%, *n* = 71). Given the ratio of males to females, we had around 75% power to detect moderate effects (differences between the two genders). Previous studies of gender differences in psychotic disorders have reported moderate to large effects [1]; thus we had an adequate sample size to power the study.

The average age of onset of illness was 22.2 years (*SD = *3.2, *min = *15, *max* = 29) and the average age at entry to the service was 22.4 years (*SD = *3.2, *min = *15, *max* = 30).

Males with FEPM were more likely to have a past history of substance use (*OR* = 4.86, *p <*.001) and forensic issues (*OR* = 14.79, *p* = .008). Females with FEPM were more likely to have experienced sexual abuse trauma (*OR* = 7.12, *p* = .001)^a^ (see Table 1).

**Table 1:** Demographic, diagnostic, premorbid, pre-treatment and service entry factors related to gender in FEPM patients

		**Gender**			**95% CI of OR**		**p value**
		**Male**	**Female**	**OR**	**LCI**	**UCI**	
**Variable**		**(n=71)**	**(n=47)**				
**Pre-treatment variables**							
Years in school	M (SD)	11.2 (1.3)	11.0 (1.6)	1.12	0.86	1.45	.395
Pre-morbid GAF	M (SD)	73.9 (11.5)	74.8 (9.4)	0.99	0.96	1.03	.638
Duration of prodrome (in days) ^a^	M (SD)	363.0 (640.0)	227.2 (338.0)	1.15	0.83	1.60	.398
Duration of untreated psychosis (in days) ^a^	M (SD)	41.4 (197.2)	64.93 (190.9)	0.76	0.50	1.16	.204
Age at onset (years)	M (SD)	22.0 (3.0)	22.5 (3.6)	0.95	0.85	1.07	.387
Past history of suicide attempt (%Yes)	% (n)	2.8 (2)	8.7 (4)	0.30	0.05	1.73	.180
Family history of psychiatric disorder	% (n)	60.9 (42)	72.1 (31)	0.60	0.26	1.37	.227
**Diagnostic variables**							
Past history							
Major depressive disorder	% (n)	49.3 (35)	63.8 (30)	0.55	0.26	1.17	.122
Substance use disorder (SUD) (%Yes)	% (n)	91.5 (65)	44.7 (21)	13.41	4.86	37.01	<.001
Sexual abuse	% (n)	5.6 (4)	29.8 (14)	0.14	0.04	0.46	.001
Physical abuse	% (n)	14.1 (10)	19.1 (9)	0.69	0.26	1.86	.465
Forensic issues	% (n)	30.4 (21)	8.5 (4)	4.71	1.50	14.79	.008
At service entry							
Comorbid psychiatric disorder (%Yes)	% (n)	4.2 (3)	4.3 (2)	1.01	0.16	6.27	.994
Substance use disorder (SUD) (%Yes)	% (n)	74.6 (53)	42.6 (20)	3.98	1.81	8.74	.001
Cannabis	% (n)	54.9 (39)	36.2 (17)	2.15	1.01	4.58	.047
Alcohol	% (n)	11.3 (8)	12.8 (6)	0.87	0.28	2.68	.805
Polysubstance	% (n)	14.1 (10)	8.5 (4)	1.76	0.52	5.99	.364
**Baseline variables**							
Age at service entry	M (SD)	22.3 (3.0)	22.7 (3.4)	0.96	0.85	1.08	.469
Symptoms at entry							
CGI-S severity score	M (SD)	5.8 (0.8)	5.5 (0.7)	1.72	1.03	2.86	.037
CGI-BP depression	M (SD)	1.5 (1.3)	1.4 (1.3)	1.11	0.81	1.51	.520
CGI-BP mania	M (SD)	4.7 (1.8)	4.6 (1.5)	1.05	0.84	1.31	.657
Insight at entry (%No)	% (n)	76.1 (54)	65.2 (30)	1.69	0.75	3.83	.205
Functional level at entry							
Employment/occupation (%Yes)	% (n)	63.4 (45)	72.3 (34)	0.66	0.30	1.47	.312
GAF	M (SD)	29.7 (10.1)	33.5 (9.1)	0.96	0.92	0.99	.045
Living with family	% (n)	54.3 (38)	44.7 (21)	1.47	0.70	3.09	.309

Note: OR - odds ratio; LCI - lower confidence interval; UCI - upper confidence interval; CGI-S - Clinical Global Impressions Scale - Severity; CGI-BP - Clinical Global Impressions Scale - Bipolar Disorder; GAF - Global Assessment of Functioning.

^a^ Raw data are presented, however the test statistics were based on log10 (+constant) transformed data because of extreme positive skewness.

At service entry, males were significantly more likely to have substance use issues (*OR* = 3.98, *p *= .001); with cannabis use prominent (*OR* = 4.58, *p* = .047). Males also had more severe illness (CGI-S, *OR* = 1.72, *p *= .037) and poorer functioning (GAF, *OR *= 0.96, *p *= .045) compared to females with FEPM (see Table 1). During treatment males with FEPM were more likely to decrease or stop their substance use (*OR *= 5.34, *p *= .008) than females with FEPM. At discharge males were more likely to be living with their families (*OR *= 4.30, *p *= .009), but there were no gender differences in discharge psychopathology or functioning (see Table 2).

**Table 2:** Treatment and discharge characteristics of males and females with FEPM

		**Gender**			**95% CI of OR**		**p value**
		**Male**	**Female**	**OR**	**LCI**	**UCI**	
**Variable**		**(n=71)**	**(n=47)**				
**Treatment variables**							
Length of time in service (in weeks)	M (SD)	64.8 (34.1)	59.1 (30.1)	1.01	0.99	1.02	.353
Admitted to hospital (%Yes) ^a^	% (n)	91.5 (65)	80.9 (38)	2.71	0.89	8.30	.080
Number of admissions ^a^	M (SD)	1.5 (1.1)	1.2 (0.8)	1.36	0.88	2.10	.173
Compliance with treatment (%Yes) ^a^	% (n)	56.5 (39)	50.0 (21)	1.28	0.59	2.77	.533
Substance use disorder (SUD) ^a b^							
No SUD	% (n)	23.9 (17)	55.3 (26)	na			
Remitted SUD (decreased or stopped)	% (n)	56.3 (40)	25.5 (12)	5.34	2.17	13.14	<.001
Persistent SUD (increased or no change)	% (n)	19.7 (14)	19.1 (9)	2.93	0.97	8.89	.056
Suicide attempt in treatment (%Yes) ^a^	% (n)	7.0 (5)	4.3 (2)	1.51	0.27	8.32	.639
Symptoms at discharge							
CGI-S severity score ^c^	M (SD)	2.2 (1.2)	2.2 (1.2)	0.99	0.69	1.42	.950
CGI-BP depression ^d^	M (SD)	1.4 (0.9)	1.4 (1.0)	0.99	0.65	1.49	.953
CGI-BP mania ^e^	M (SD)	1.4 (1.0)	1.5 (1.0)	1.03	0.65	1.64	.890
Insight at discharge (%No) ^f^	% (n)	7.0 (5)	4.3 (2)	1.88	0.32	11.14	.485
Functional level at discharge							
Employment/occupation (%Yes) ^g^	% (n)	56.5 (35)	72.5 (29)	0.50	0.19	1.31	.158
GAF ^h^	M (SD)	68.8 (14.6)	71.1 (12.3)	0.99	0.96	1.02	.446
Living with family (%Yes) ^'^	M (SD)	77.0 (47)	51.3 (20)	4.30	1.44	12.79	.009

Note: OR - odds ratio; LCI - lower confidence interval; UCI - upper confidence interval; CGI-S - Clinical Global Impressions Scale - Severity; CGI-BP - Clinical Global Impressions Scale - Bipolar Disorder; GAF - Global Assessment of Functioning.

^a^ Covariate was time in service.

^b^ No substance use was reference category in logistic regression.

^c^ Covariates were time in service and CGI-S at entry, unadjusted means and standard deviations are reported.

^d^ Covariates were time in service and CGI-BP depression at entry, unadjusted means and standard deviations are reported.

^e^ Covariates were time in service and CGI-BP mania at entry, unadjusted means and standard deviations are reported.

^f^ Covariates were time in service and insight at service entry.

^g^ Covariates were time in service and employment status at entry.

^h^ Covariates were time in service and GAF score at entry.

^i^ Covariates were time in service and living with family at service entry.

A range of variables in the univariate analyses distinguished males and females at the *p* < .100 level including: past history of substance use; exposure to sexual abuse; forensic issues; substance use at service entry; CGI-S at entry; GAF at entry; admissions to hospital; substance use at discharge; and whether or not living with a family at discharge. These variables were entered into a multivariate logistic regression model to determine which factors best delineated differences between males and females. Past history of substance use (*OR* = 20.33, 95% CI *OR* 6.13-67.42, *p *< .001), exposure to sexual abuse (*OR *= 0.11, 95% CI *OR* 0.02-0.53, *p *= .006), and living with family at discharge (*OR *= 3.33, 95% CI *OR* 1.06-10.41, *p *= .039) were the variables that best discriminated between males and females with FEPM.

## Discussion

To our knowledge, this the first study to comprehensively examine gender differences in illness onset and course in a treated epidemiological cohort of patients with FEPM. A further strength of the study is that we included all patients who first presented for treatment and not just those patients with more severe or chronic illness or those who have had their first inpatient admission. Similar to previous studies into patients with chronic illness and bipolar I disorder, we found an array of gender differences in patients with FEPM between the ages of 15 to 29 years. Key differences were found with respect to premorbid history, severity of illness at entry, treatment and discharge characteristics.

Univariate analyses indicated that males with FEPM were more likely to have a history of substance and forensic issues. It has previously been shown that males with bipolar disorder are more likely to have conduct problems or antisocial tendencies in childhood [7,8] which may increase the likelihood of violence [31] and problems with the law [14,32]. Substance use (especially, alcohol, cannabis and nicotine) also tends to be more common in males than females with bipolar disorder [9,10,15,33], and might explain the increased prevalence of forensic issues [34] in our cohort.

Gender differences in the prevalence of substance use disorders have been reported in the general population, with males being more likely to abuse or be dependent on drugs [35]. At service entry, 74.6% of males and 42.6% of females were using substances; much higher than rates in the general population. The persistent substance use in males, particularly prior to, and at the onset of the psychotic mania, might in part explain their more severe levels of psychopathology and poorer functioning (as measured by the GAF). This is supported by findings that substance use in patients with bipolar disorder is associated with poorer social adjustment and outcome [36].

Males were more likely to use cannabis than females with FEPM; but no such gender differences were found with alcohol or polysubstance use. Cannabis use is common in patients with bipolar disorder, particularly in younger patients (< 30 years) [37,38]. Cannabis use may be associated with an earlier age of onset of bipolar disorder, particularly in vulnerable individuals [38]. However, no such gender differences in age of onset were found in the current study cohort.

During treatment, males were more likely than females to decrease or cease their substance use. Increased familial support in males might have contributed to the reduction of substance use. Males who are ill with a first episode of psychosis are more likely than females to receive understanding and support from family members [39]. This may relate to cultural and societal differences in that underperformance and behavioural issues are tolerated more in males than females [39]. Further, females with psychotic disorders such as schizophrenia are more likely to have experienced childhood adversities [40], and are hence likely to leave home at an early age. Family involvement in early treatment can be pertinent for minimising the chances of substance use becoming a more serious disorder [41-43]. An alternative explanation might be that the percentage of males who live with their families increases when they stop using substances. The decrease in substance use in males may also account for why there are no differences between the genders in terms of severity of illness and functioning at discharge.

Females were seven times more likely to have experienced sexual abuse than males with FEPM. A recent meta analysis indicated that the prevalence of sexual abuse is higher in females (19.7%) than males (7.9%) in the general population [44]. However, in our sample approximately 30% of females with FEPM had been exposed to sexual abuse; a rate of trauma that is of concern. A relationship between sexual abuse or traumatic experiences and the onset of psychotic disorders has been proposed [45-48], and this association may be stronger in females than males [49]. Exposure to such trauma, may negatively affect brain development, through chronic hyper-reactivity of the hypothalamic-pituitary-adrenal (HPA) axis. This hyper-reactivity may then lead to disruption in the functions of dopaminergic and serotonergic systems, and may worsen prognosis. Such events sequelae have been described in the “traumagenic neurodevelopmental” model. Although the ultimate alterations to functions in dopaminergic and serotonergic may precipitate the onset of FEPM, there may be broader long-term effects. In females with chronic bipolar disorder, a history of sexual and physical abuse/trauma may relate to poorer mood outcomes such as more shifts in polarity and mixed episodes [50]. Exposure to sexual abuse might also place females with bipolar disorder at a disproportionately greater risk of comorbid anxiety disorders such as post-traumatic stress disorder (PTSD) [14,47,48,51]. Because our cohort was young, the possibility of the development of such (generally) longer-term sequelae might not have been captured in this group.

A history of sexual abuse may also explain why females with FEPM were less likely to be living with their families at discharge from the service. If the abuse occurred within the context of the family networks, then this is a unsurprising finding. However, it should also be noted, a history of sexual abuse and/or trauma is linked with markedly poorer social functioning especially with respect to building constructive and trusting relationships [52].

With regards to the characteristics of the current study, a number of factors need to be kept in mind in interpreting the data. FEPM cohorts may offer a unique window into the disorder, as the effects of illness progression and treatment are controlled for. The primary limitation of this study is the use of a retrospective medical file audit. There can be numerous problems with such an approach including: (i) poor quality of documented information; (ii) clinical experience of raters; (iii) lack of inter-rater reliability; and (iv) questionable data validity. Numerous strategies were adopted in the current study to minimise the impact of such limitations including: (i) medical files at EPPIC were systematised according to FEP guidelines [53]; (ii) medical files were rated by two consultant psychiatrists with expertise knowledge of EPPIC and the treatment of FEP; (iii) sound inter-rater reliability was determined for clinical and functioning measures; and (iv) concurrent validity of psychoses and baseline SUD was established for a sub-sample of patients [20]. The study population was a catchment area sample, and is likely to be broadly representative of the population of FEPM (16).

A further study limitation was the lack of information recorded regarding psychopathology (e.g., mood episodes, psychotic symptoms, relapse) and type and dosage of pharmacological treatments; these factors could have had an effect on the degree of gender difference at discharge.

## Conclusions

It appears that there are substantial differences between males and females with FEPM in terms of past histories, presentation, and discharge outcomes; however, these differences may be driven by putative risk factors such as substance use and exposure to sexual abuse. Importantly, these risk factors occur prior to the onset of full threshold disorder. These risk factors might be markers of a disease that has already been under development for some time. However, given current evidence, it is difficult to ascertain whether substance abuse and/or sexual abuse are a cause or consequence (e.g., prodromal phase of illness) of illness.

There is growing interest in identifying individuals at “ultra high risk” of developing bipolar disorder [54] and who have a clearly defined prodrome [55,56]. It has been argued that symptoms alone are unlikely to be sufficient for the correct identification of those at risk of developing bipolar disorder; however, identifying risk factors or markers of vulnerability may improve the accuracy of this prediction [55]. The substance use and sexual abuse findings from the current study may be considered such gender specific risk factors that increase the likelihood of developing FEPM and bipolar disorder. Such information could be used by clinicians working with such “high risk” individuals to identify those who might be at the greatest risk of developing first psychotic manic episode. Future research could also be directed to whether there are gender differences in pharmacological response in patients who are either at high risk or have full threshold disorder.

## Endnote

^a^For ease of interpretation, female was the reference category for the calculation of this odds ratio.

## Abbreviations

FEP: First episode psychosis; FEM: First episode mania; FEPM: First episode psychotic mania; FEPOS: First Episode Psychosis Outcome Study; EPPIC: Early Psychosis Prevention and Intervention Centre; DSM-IV: Diagnostic and Statistical Manual of Mental Disorders – fourth edition; ICD-10: International Classification of Disease – 10^th^ edition; CGI-S: Clinical Global Impressions Scale – Severity; CGI-BP: Clinical Global Impressions Scale – Bipolar Disorder; GAF: Global Assessment of Functioning; MVSI: Modified Vocation Status Index; MLCI: Modified Location Code Index; OR: Odds ratio; CI: Confidence interval

## Competing interests

Prof. Lambert, Prof. Conus, Prof. Schimmelmann, and Prof. McGorry have served on the speakers’ board of Eli Lily. Prof Berk Berk has served on advisory boards and received funds and/or honoraria from Astra Zeneca, Eli Lilly, Janssen Cilag, Lundbeck, Servier, Glaxo Smith Kline, Organon, Novartis, Pfizer, Bristol Myers Squibb, Sanofi Synthelabo and Solvay.

There are no other relevant disclosures.

## Authors’ contributions

ML, PC, and PM contributed to the design and conduct of FEPOS. SC and MB developed the ideas for this paper. SC wrote the draft of the manuscript and conducted all statistical analyses. ML, PC, PM, FJB, and BGS collaborated in writing and editing of the paper and approved the final manuscript.

## Pre-publication history

The pre-publication history for this paper can be accessed here:

http://www.biomedcentral.com/1471-244X/13/82/prepub
